# Mechanisms of Enhanced Thermal Durability in Porosity-Controlled Multilayer Thermal Barrier Coatings

**DOI:** 10.3390/ma18050917

**Published:** 2025-02-20

**Authors:** Janghyeok Pyeon, Kyung-Moo Kang, Bong-Gu Kim, Jeonghyeon Lee, Sohee Baek, Seungcheol Yang, Yeon-Gil Jung, Dowon Song, Byungil Yang

**Affiliations:** 1 Department of Materials Convergence and System Engineering, School of Materials Science and Engineering, Changwon National University, Changwon 51140, Republic of Koreapure5258@changwon.ac.kr (S.Y.);; 2 Sung-il Turbine P&S, Gangseo-gu, Busan 46753, Republic of Korea; 3 School of Materials Science and Engineering, Kumoh National Institute of Technology, Gumi 39177, Republic of Korea

**Keywords:** thermal barrier coatings, multilayer, gradient coatings, thermal cycling, microstructures

## Abstract

This study investigates the enhancement of thermal durability in multilayer yttria-stabilized zirconia (YSZ) thermal barrier coatings (TBC) with porosity-controlled structures. Conventional single-layer YSZ and multilayer TBCs with dense and porous layers were fabricated by air plasma spraying and the TBC specimens were subjected to furnace cyclic testing. The single-layer TBC suffered from catastrophic delamination under cyclic thermal loading, driven by the mismatch in thermal expansion, while the multilayer TBCs exhibited a significant increase in thermal durability, by up to 50%. The relevant delamination mechanism was suggested with microstructural analysis, showing that the multilayer structure effectively relieved residual stresses by forming horizontal cracks, thereby mitigating crack propagation. This study emphasizes that the multilayer design in TBC with controlled porosity significantly enhances thermal durability, improving the operational lifespan of gas turbine hot components.

## 1. Introduction

A gas turbine is a thermal engine that burns a mixture of compressed air and fuel to produce high-temperature and high-pressure combustion gas [1,2,3]. These combustion gases expand, rotating the turbine blades’ mechanical power or changing this energy into electricity via a generator. Gas turbines are used in a variety of fields, such as the aerospace, aviation, and marine industries, which has led to numerous research and development efforts [4,5].

In order to increase the fuel efficiency of gas turbines for power generation, research is focused on increasing the turbine inlet temperature (TIT), which is the high temperature point of the Carnot cycles [6,7]. To achieve a higher TIT, leading gas turbine manufacturers have introduced advanced technologies, such as superalloy, internal cooling channels, and thermal barrier coating (TBC) [8,9]. These advancements enable turbines to operate at higher temperatures and, ultimately, to achieve superior thermal efficiency.

Gas turbines for power generation typically manage peak loads rather than continuous baseload operations, resulting in frequent start-stop cycles [10,11]. This periodic operation exposes the hot components to severe environments with high temperature and high pressure, including thermal fatigue, oxidation, corrosion, and erosion [12,13]. To reduce damage under these challenging conditions, TBCs are applied to hot components to improve both operating temperatures and thermal durability.

TBCs are deposited on the surface of a superalloy and consist of a ceramic top coat with low thermal conductivity, and a bond coat which can enhance adhesion between the ceramic top coat and metallic substrate. As the turbines operate, these coatings are exposed to the abovementioned environments. Therefore, the top-coat material should possess sufficient stability to endure thermal, mechanical, and chemical stress. Yttria-stabilized zirconia (YSZ) has been typically used for top-coat material due to its low thermal conductivity, high melting point, phase stability, and compatibility with metallic substrates owing to its relatively high thermal expansion coefficient [14,15,16].

As previously mentioned, gas turbines suffer from frequent start-stop cycles, resulting in TBC spallation, which leads to considerable costs associated with maintenance, repair, and replacement. To overcome these issues, many studies have been aimed at modifying the structure of traditional YSZ coatings or developing TBCs with new compositions [17,18,19]. Previous reports aimed at enhancing the thermal durability of conventional YSZ-TBCs have introduced dense vertically cracked (DVC) structures [20,21,22,23]. A DVC-TBC is a high-density coating layer which develops vertical cracks during cooling after thermal spraying, absorbing the stresses induced by the thermal expansion mismatch between the top coat and the substrate during start-stop cycles, and thereby improving thermal durability. These DVC-TBCs can be applied in thicknesses of up to 2000 μm on non-rotating components with relatively lower mechanical stress, such as parts of the combustor.

Furthermore, multilayer structures and functionally graded coatings have been investigated for the application of new candidate top-coat materials [24,25,26]. Although these innovative materials may provide superior thermal insulation compared to conventional YSZ, they often exhibit inferior thermal expansion coefficients, mechanical properties, and particularly fracture toughness, resulting in poor thermal durability compared to conventional YSZ-TBCs. To address these challenges, multilayer coatings with YSZ as a buffer layer or composite structures with various graded compositions have been suggested, achieving enhanced thermal durability comparable to conventional YSZ-TBCs [27]. Other studies have suggested an optimized structure with gradually designed porosity and density.

Several studies have investigated the influence of porosity in single-layer TBCs. Computational investigation of porosity effects on the fracture behavior of TBCs has been conducted, suggesting that porosity plays a significant role in improving the fracture resistance of single-layer TBCs [28]. Yantong et al. recently revealed that higher porosity relieves the thermal expansion mismatch stress within thick TBCs [29]. However, research on controlling porosity and thermal durability in multilayer TBCs remains limited.

This study investigates multilayer YSZ-TBCs with controlled porosity to optimize TBC structures with a thickness exceeding 500 μm. TBC specimens were fabricated via plasma spraying using different feedstock powders, with layer thicknesses adjusted to achieve controlled porosity of less than 10% in the dense layer and up to 20% in the porous layer. The thermal durability of the TBCs was evaluated via furnace cyclic tests. The multilayer coatings showed enhanced thermal durability compared to conventional single-layer TBCs. Additionally, through an analysis of failure mechanisms within multilayer TBCs, this study highlights the potential for further enhancement of thermal durability through targeted porosity and thickness optimization in multilayer YSZ-TBC systems.

## 2. Experimental Procedure

### 2.1. Sample Preparation

The coin-shaped Ni-based superalloy (Nimonic 263, ThyssenKrupp VDM, Werdohl, Germany, nominal composition of Ni–20Cr–20Co–5.9Mo–0.5Al–2.1Ti–0.4Mn–0.3Si–0.06C, in wt.%) was used as a metal substrate for the multilayer TBC specimens, with dimensions of 25 mm in diameter and 5 mm in thickness. Prior to the application of the TBC, the substrate surface was sandblasted to optimize the roughness, enhancing the adhesion of the coating. After sandblasting, any contaminants, such as oils and dust, were cleared using compressed air and a soft brush.

A nickel-based metal powder (AMDRY 386-3, Oerlikon Metco, Pfäffikon, Switzerland) was applied to fabricate the bond coat layer, achieving a thickness of around 150 μm through the high-velocity oxygen fuel (HVOF) spraying technique. Due to the low surface roughness resulting from the high velocity of feedstock powder in the HVOF process, a flash coating procedure employing atmospheric plasma spraying (APS) was used to enhance the surface roughness and adhesion strength. The detailed coating parameters are presented in Table 1.

**Figure 1:**
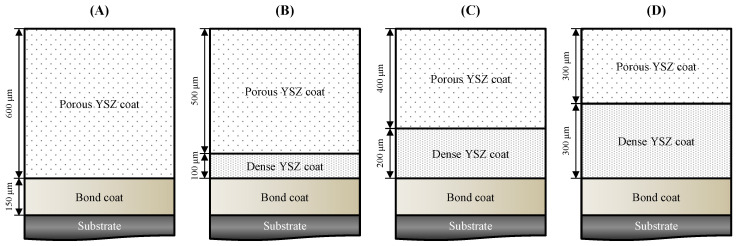
Schematic diagram of microstructure design for the single- and double-layer TBCs with different porosities. (**A**) Single-layer TBC, (**B**) Multilayer TBC with a 100 µm thickness of high-density layer, (**C**) Multilayer TBC with a 200 µm thickness of high-density layer, and (**D**) Multilayer TBC with a 300 µm thickness of high-density layer.

After the bond coat fabrication, a YSZ-based feedstock was used for the preparation of multilayer TBCs. To control the porosity in each layer, two types of commercial YSZ feedstocks (Metco 204AF and Metco 204-NS P, Oerlikon Metco, Switzerland) were applied; their chemical composition and particle size are detailed in Table 2. The multilayer top coats were deposited using the APS (9 MB; Oerlikon Metco, Switzerland) method with porosity levels of less than 10% for dense layers and up to 20% for porous layers. The structure of the designed TBC specimens, along with the desired thickness and porosity specifications for each layer, is illustrated in Figure 1. The targeted thickness and porosity for the single- and double-layer TBCs are presented in Table 3.

### 2.2. Characterizations and Thermal Durability Tests

The morphology and microstructure of feedstock powder were analyzed with a scanning electron microscope (SEM, JSM5610, JEOL, Tokyo, Japan), and a particle size distribution analysis was conducted for both powders. To investigate both the powder morphology and the cross-sectional microstructure before and after the thermal durability tests, the feedstock powders and each TBC specimen were cold-mounted using epoxy resin. The mounted specimen was cut and polished using silicon carbide paper and the 3 and 1 μm diamond pastes, respectively. The thermal durability of the TBCs was assessed through furnace cyclic tests (FCT). The thermal cycling protocol for evaluating durability involved heating from 20 °C to 1100 °C for 15 min, a dwell time for 25 min, and then cooling back to 20 °C for 20 min, with each cycle repeated until spallation was observed in the TBC specimens. Failure of TBCs was defined by spallation of over 30% of the top-coat region or cracking at the interface between the top and bond coat. The microstructure of the TBC specimens was examined using SEM before and after the thermal durability tests. The porosity was analyzed using the ImageJ software (ImageJ version 1.8.0, NIH, USA).

## 3. Results and Discussion

### 3.1. Analysis of Powders and Fabricated TBC Specimens

**Figure 2:**
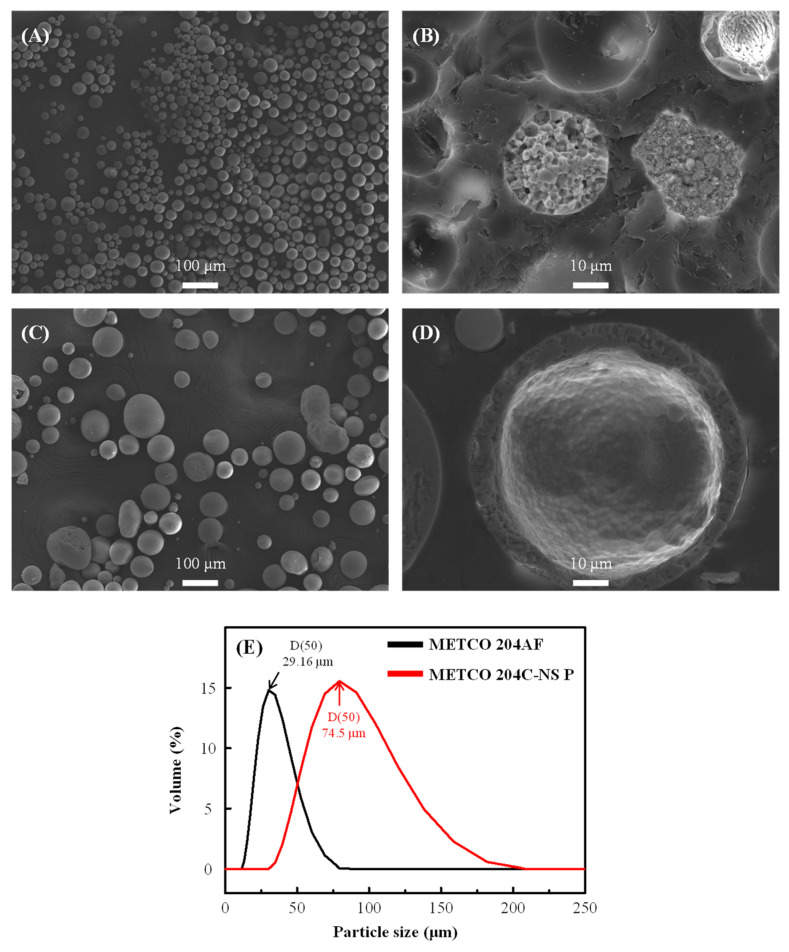
Microstructure and particle size distribution for (**A**,**B**): METCO 204AF, (**C**,**D**): METCO 204 C-NS Premium and (**E**) Particle size distribution.

The microstructure analysis and particle size distribution of the two commercial YSZ feedstocks (Metco 204-NS P and Metco 204AF for the fabrication low- and high-density layers, respectively) are shown in Figure 2. The average particle size of the powder for the high-density layer measured 29.16 μm, which is notably smaller than the low-density powder particle size of 74.50 μm. The high-density powder is primarily made up of densely packed particles with very little internal porosity. This property reduces the number of internal pores and the splat boundaries which developed at the interfaces between the feedstock splats during the plasma spraying process, limiting potential pathways for crack propagation [30,31].

On the other hand, the low-density powder has larger particle sizes than the high-density powders, containing hollow sphere (HOSP) particles. This microstructure results in a higher porosity in the coating layer and a larger number of splat boundaries, which is anticipated to enhance thermal barrier performance and stress accommodation within the coating layer [32,33].

**Figure 3:**
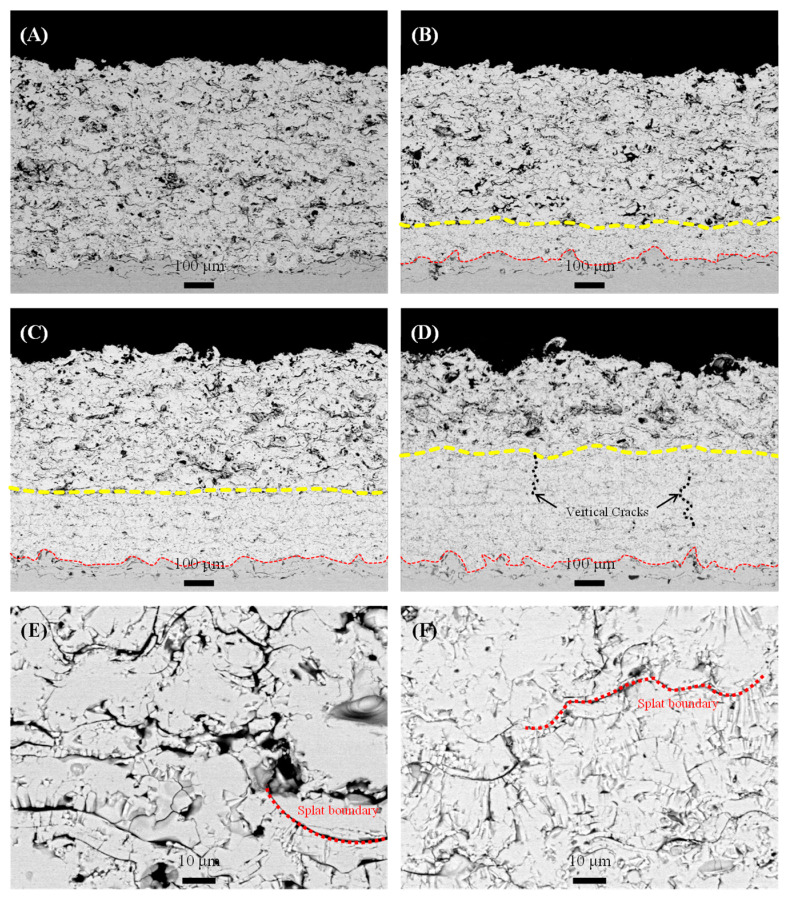
Microstructures of as-coated specimens: (**A**) sample A, (**B**) sample B, (**C**) sample C and (**D**) sample D. Highly magnified cross-sectional microstructures: (**E**) porous layer and (**F**) dense layer in sample D.

Figure 3 shows the cross-sectional microstructure of both single-layer and multilayer TBC specimens, implying successful fabrication with a thickness of around 500 μm in all specimens. Figure 3B–D indicate that high-density layers with target thicknesses of approximately 100, 200, and 300 μm were appropriately prepared between the bond coat and the low-density top-coat layer. The interface low-/high-density coating and YSZ/bond coating interface were marked by yellow and red dashed lines, respectively, in Figure 3B–D. The measured porosity for the low- and high-density layers were 17.1 ± 2.4% and 11.6 ± 2.1%, respectively, in accordance with the desired porosity. However, the high-density layer exhibited slightly enhanced porosity compared to the target 10%, which was attributed to vertical cracks that developed during the cooling process [34,35,36]. These cracks are considered to enhance thermal durability by accommodating the horizontal stress resulting from the mismatch in thermal expansion coefficients between the top coats and the substrate.

Figure 3E,F provide enlarged views of the microstructure in the low- and high-density coating layers, respectively. A layered structure can be seen, characterized by splat boundaries, which are an interface between feedstock splats formed through the plasma spraying process, as well as accompanying microcracks and pores generated during cooling after melting [37]. The microstructure analysis reveals a sound condition of the TBC structure without significant process-induced defects, such as large cracks or horizontal cracks at the low-/high-density coating interface or the YSZ/bond coating interface.

### 3.2. Enhancement of Thermal Durability Through Multilayer Structure and Porosity Control

**Figure 4:**
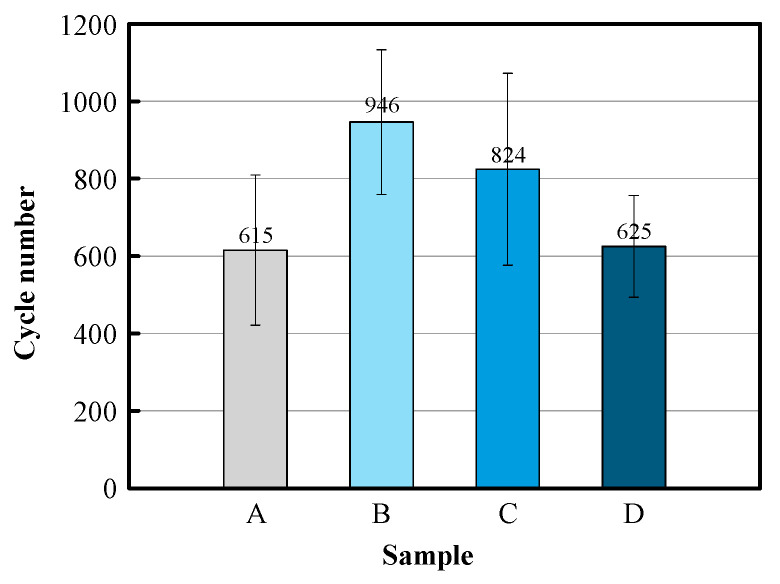
Comparison of numbers of cycle-to-failure for thermal durability test.

The thermal durability of the specimens prepared was evaluated through furnace cyclic testing (FCT) and at least four samples were tested for each specimen. Figure 4 shows the average lifespan of four specimens for each coating specimen. Specimens B and C, with double-layer structures, demonstrated overall enhanced thermal durability compared to specimen A, which consisted of a single low-density layer. Notably, specimens B and C, which included high-density layers of 100 μm and 200 μm thickness, exhibited an improvement in thermal durability of about 30–50%.

**Figure 5:**
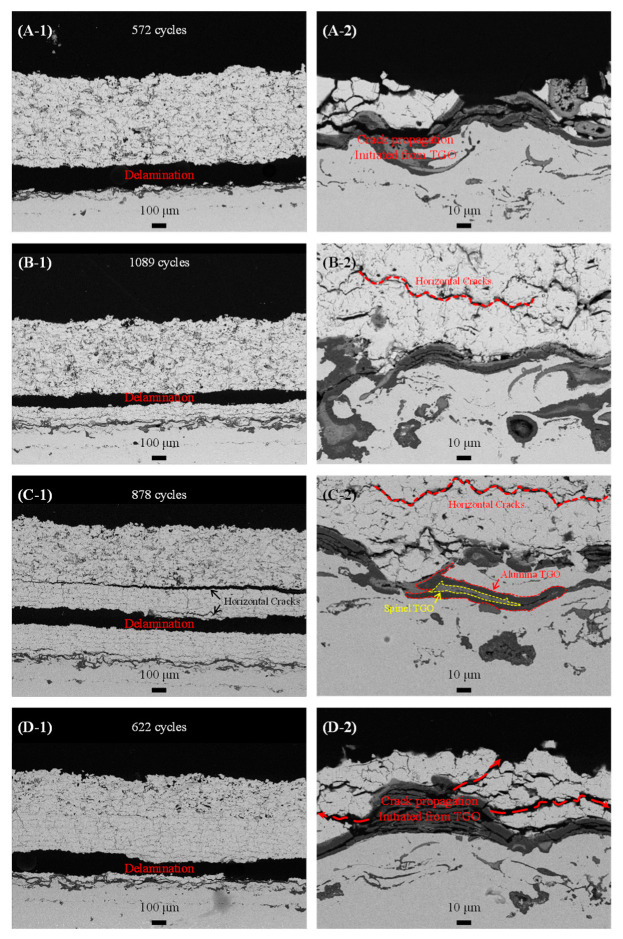
Cross-sectional microstructure after FCT tests: (**A**) sample A, (**B**) sample B, (**C**) sample C and (**D**) sample D. The numbers of cycle-to-failures for each figure is shown inside each figure. Each number indicates the low-magnified microstructures and high-magnified interface microstructure, respectively.

Figure 5 shows the cross-sectional microstructures of each specimen after the FCT, providing an evaluation of the influence of the multilayer structure and porosity control on thermal durability. The delamination regions and the number of FCT cycles for each specimen were included, with the right-side images exhibiting enlarged microstructures at the YSZ-bond coat interface, where stress was most concentrated during the FCT.

In the microstructures observed in a low magnification image (Figure 5(A-1,D-1)), it was apparent that for specimen A (single-layer) and specimen D (with a high-density layer constituting half of the total thickness), there was complete delamination of the YSZ layer within approximately 100 μm above the bond coat surface. In contrast, for specimen B, where the high-density layer composed nearly 15% of the coating thickness, delamination was seen in the 100–200 μm range from the interface, specifically occurring at the interface between the low- and high-density layers (Figure 5(B-1)). Similarly, in specimen C, where the high-density layer made up approximately 33% of the total thickness, delamination was observed within the 200–300 μm region and showed numerous horizontal cracks similar to those in specimen B (Figure 5(C-1)). Moreover, significant horizontal cracks formed at the interface between the low- and high-density layers, directly contributing to the observed delamination. The residual high-density layers in specimens B and C contained horizontal cracks, although these cracks did not propagate to cause delamination [38,39].

In the enlarged microstructures of specimens A and D, a thermally grown oxide (TGO) layer was observed, measuring about 16 μm and 15 μm thick, respectively. This layer was produced due to high-temperature exposure to oxygen during cyclic testing. Cracks within the TGO layer were directly associated with delamination, a failure mechanism frequently seen in specimen A. In the early phases of the FCT, the metal components in the bond coat, especially aluminum, oxidized and formed a thin and dense alumina layer [40]. This layer suffered from severe thermo-mechanical stress and eventually fractured, promoting additional alumina growth at the TGO-bond coat interface during FCT cycles (see Figure 5(A-2,D-2)). The continuous formation and failure of the alumina TGO layer resulted in cracks that interconnected with delaminated regions in specimens A and D. This suggests that thermal stresses from the cooling and heating cycles caused cracks within the alumina TGO, which eventually resulted in the delamination of the coating layer [41].

As the TGO layer continued to grow, aluminum was depleted in certain areas of the bond coat [42,43]. In the aluminum-depleted regions, spinel-type TGO structures, such as Mal_2_O_4_ (with M representing elements like Ni, Cr, Co, etc.), were formed, as verified in Figure 5(B-2,C-2). The average TGO thicknesses in specimens B and C were 26 μm and 23 μm, respectively, showing larger thickness compared to specimens A and D due to longer FCT cycles, coordinating with the increased thermal durability shown in Figure 4.

Meanwhile, horizontal cracks were observed near the YSZ-bond coat interface in specimens B and C, as illustrated in Figure 5(B-1,C-1). In contrast, these cracks were largely absent in specimens A and D (see Figure 5(A-1,D-1)). The horizontal cracks found in the remaining coatings of specimens B and C did not directly contribute to delamination. Instead, these cracks served to disperse crack propagation paths and relieved thermal stresses within 100 μm from the interface through various crack formations, contributing to an improved thermal durability [44]. The improvement in thermal durability through crack dispersion is further evidenced by the critical thickness of the TGO, which is typically below 10 μm under cyclic high-temperature exposure tests [45,46,47].

In specimens A and D, where stresses were concentrated within 100 μm of the interface, cracks emerged within the TGO and were linked to delamination [48]. However, even though the TGO growth in specimens B and C further exceeded the critical thickness, the cracks that developed within the TGO did not connect directly to delamination. Instead, horizontal cracks developed above the interface, promoting stress dispersion and finally resulting in superior thermal durability [49].

### 3.3. Analysis of Mechanisms for Enhanced Thermal Durability

**Figure 6:**
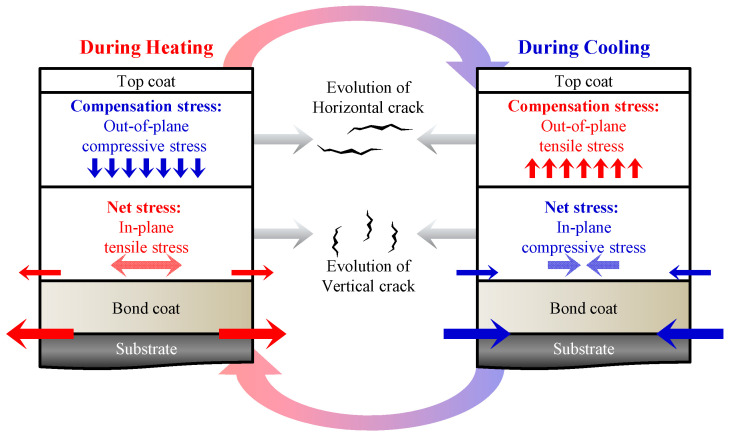
Schematics of stress distribution and crack evolution within the YSZ top coat during cyclic thermal exposure.

This section investigates the effect of a porosity-controlled multilayer structure on thermal durability. Figure 6 illustrates the stress distribution and mechanisms of crack formation during the FCT process. During the heating process, the YSZ layer suffers from the in-plane tensile stresses, which are generated due to its relatively low coefficient of thermal expansion compared to the metal substrate and bond coat [17,19]. Consequently, it induces compensatory out-of-plane compressive stresses. On the other hand, during the cooling process, in-plane compressive stresses and out-of-plane tensile stresses occur within the YSZ layer. The intensity of these stresses is greatest at the YSZ-bond coat interface, decreasing with distance from that interface. After the cooling process, the thermal stresses induced are retained as residual stresses near the YSZ layer. With repeated heating and cooling cycles, plastic deformation occurs, relieving part of the accumulated stress. In-plane stress primarily promotes vertical crack growth, whereas out-of-plane stress mainly drives horizontal crack growth [50,51].

**Figure 7:**
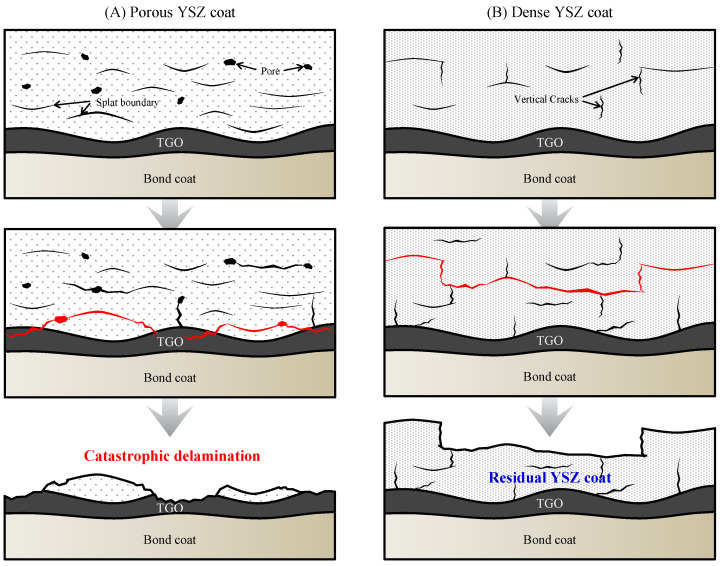
Illustration of the delamination behavior in (**A**): porous and (**B**): dense YSZ coats during thermal cycling, respectively.

Based on the stress gradient and crack formation shown in Figure 6, this provides the detailed progression from the initial microstructure through crack initiation and propagation under thermal loads, ultimately leading to delamination. In low-density YSZ coatings (Figure 7A), various initial defects, including splat boundaries and large pores, form voids that can reduce the thermal conductivity. These voids cause localized stress concentrations due to high temperature gradients at the edges of each defect [52].

As thermal cycling continues, these thermal gradients promote stress relaxation with plastic deformation and crack propagation which can relieve the residual stress. Additionally, significant stress concentrations are observed within the TGO layer, which forms along the bond coat interface. Due to its lower thermal expansion coefficient compared to YSZ and its location, fractures in the TGO layer are directly associated with delamination of TBCs. Ultimately, repeated thermo-mechanical stress in low-density coatings leads to severe delamination, resulting in complete detachment and potential oxidation of the bond coat, which ultimately damages the metallic substrate. In contrast, high-density YSZ coatings (Figure 7B) exhibit vertical cracks formed during cooling. These coatings contain fewer splat boundaries and large pores, which contribute less to delamination compared to low-density coatings. As thermal cycling continues, stress concentration occurs at the ends of defects in both low-density and high-density coatings.

However, in high-density coatings, vertical crack growth deflects horizontally due to the layered splat structure, resulting in the formation of horizontal cracks. These horizontal cracks can connect with pre-existing vertical cracks, causing stress concentration to refract vertically again with less frequency than the initial refraction. Figure 5(B-2,C-2) demonstrate that this refraction produces horizontal cracks at multiple points from the YSZ-bond coat interface, effectively distributing thermal stress through extensive plastic deformation. Horizontal crack growth mainly occurs at the interface between high- and low-density YSZ layers, significantly relieving stress as thermal cycling continues and thereby enhancing thermal durability [53,54].

As shown in Figure 5(B-1), delamination occurred at the high-/low-density YSZ interface in specimen B, with horizontal cracks observed within the residual high-density YSZ layer. Specimen C exhibited extensive horizontal cracking and larger cracks at the high-/low-density interface, indicating comprehensive stress relief, which is essential for improved thermal durability. In specimen D, however, the high-density layer extended up to 300 μm from the YSZ-bond coat interface, where stress was most concentrated. As a result, out-of-plane stresses remained within the high-density YSZ layer, and the lack of horizontal crack growth at the high-/low-density layer interface—where stress relief predominantly occurs—led to relatively poor thermal durability, as shown in Figure 5(D-1).

It is important to note that the coating’s structural design—particularly in relation to factors such as the thickness of the high-density layer—plays a crucial role in determining the thermal durability of multilayer TBCs. Li et al. suggested that large residual stress appears approximately 100 μm above the YSZ-bond coat interface, and its specific drop is observed beyond 200 μm [55]. As discussed in specimens B–D, the experimental results are consistent with the observed trend of decreasing residual stresses. This consistency highlights the necessity of optimizing the dense layer thickness to more effectively regulate residual stresses and mitigate catastrophic delamination, rather than considering only microstructural porosity control.

Ultimately, specimens B and C exhibited delamination behavior with residual coating located above the interface due to their optimally controlled porosity and layer thickness in their multilayer structures. This behavior occurred instead of catastrophic delamination caused by cracks in the TGO layer, even after undergoing thermal cycling (Figure 5(B-1,C-1)). The residual YSZ layer, with a thickness of 200 to 300 μm, continued to protect the substrate despite further thermal cycling following delamination. This observation indicates the potential for achieving both high thermal durability and minimized substrate damage during high-temperature component repair and maintenance, providing a promising approach to extending the operational lifespan of hot components.

## 4. Conclusions

This study demonstrated that thermal durability can be enhanced by employing a porosity-controlled multilayer TBC structure, suggesting the following key findings:

- Employing porosity-controlled multilayer TBCs improved thermal durability by up to 50% compared to single-layer coatings, especially with a high-density layer within 200 µm.
- Analysis of the stress distribution during thermal cycling provided insights into the delamination mechanisms occurring in both high- and low-density YSZ layers.
- Optimizing high-density layer thickness to regulate residual stresses and prevent catastrophic delamination is crucial for achieving high thermal durability in multilayer TBCs.
- Owing to the residual YSZ layer, better thermal durability and minimal substrate damage can be achieved during high-temperature repairs, offering a promising way to extend the lifespan of hot components.

## Figures and Tables

**Table 1:** Processing parameters for the preparation of TBC specimens.

Item	Gun	Feeding Rate	Gun Distance	Gun Speed	Step Distance	Carrier Gas	Flow Rate
Bond coat (HVOF)	DJ2600	60 ± 1 g/min	350 ± 5 mm	150 ± 50 mm/s	4 mm	Ar	30 ± 2 L/min
Top coat (APS)	9MB	50 ± 5 g/min	110 ± 30 mm	250 ± 50 mm/s	5 mm	N2	15 ± 1 L/min

**Table 2:** Chemical composition and particle size of coating powder.

Coating Powder	Chemical Composition						Particle Size
	Y_2_O_3_	SiO_2_	TiO_2_	Al_2_O_3_	Fe_2_O_3_	ZrO_2_	Nominal Range, μm
Metco 204C−NS P (Porous layer)	7−8	0.05	0.05	0.05	0.05	Bal.	−145 + 45
Metco 204AF (Dense layer)	7−8	0.3	0.2	0.2	0.2	Bal.	−45 + 15

**Table 3:** Targeted thickness and porosity for the single- and double-layer TBCs.

Sample	Bond Coat	Dense Layer		Porous Layer	
	Thickness (μm)	Thickness (μm)	Porosity (%)	Thickness (μm)	Porosity (%)
(A)	150 ± 30	-	-	600	~20
(B)	150 ± 30	100 ± 30	~10	500 ± 30	~20
(C)	150 ± 30	200 ± 30	~10	400 ± 30	~20
(D)	150 ± 30	300 ± 30	~10	300 ± 30	~20

## Data Availability

The original contributions presented in the study are included in the article, further inquiries can be directed to the corresponding author.
